# Evaluation of the Precision of Kinetic Stem Cell (KSC) Counting for Specific Quantification of Human Mesenchymal Stem Cells in Heterogeneous Tissue Cell Preparations

**DOI:** 10.3390/life14010051

**Published:** 2023-12-28

**Authors:** Hitesh Chopra, Michael P. Daley, Adhya Kumar, James Sugai, Alex Dahlkemper, Darnell Kaigler, James L. Sherley

**Affiliations:** 1 Department of Periodontics and Oral Medicine, School of Dentistry, University of Michigan, Ann Arbor, MI 48109, USA; choprah@umich.edu (H.C.); jsugai@umich.edu (J.S.); abdahlkemper@gmail.com (A.D.); dkaigler@umich.edu (D.K.); 2 Institute for Applied Life Sciences, University of Massachusetts, Amherst, MA 01003, USA; mpdaley@umass.edu; 3 Drik LLC, Oklahoma City, OK 73104, USA; info@atdrik.com; 4 Department of Biomedical Engineering, University of Michigan, Ann Arbor, MI 48109, USA; 5 Asymmetrex^®^ LLC, Boston, MA 02130, USA

**Keywords:** tissue stem cell, mesenchymal stem cell, cell therapy, stem cell fraction, tissue cell kinetics, alveolar bone, kinetic stem cell counting, rapid counting algorithm, precision, variation

## Abstract

Kinetic stem cell (KSC) counting is a recently introduced first technology for quantifying tissue stem cells in vertebrate organ and tissue cell preparations. Previously, effective quantification of the fraction or dosage of tissue stem cells had been largely lacking in stem cell science and medicine. A general method for the quantification of tissue stem cells will accelerate progress in both of these disciplines as well as related industries like drug development. Triplicate samples of human oral alveolar bone cell preparations, which contain mesenchymal stem cells (MSCs), were used to estimate the precision of KSC counting analyses conducted at three independent sites. A high degree of intra-site precision was found, with coefficients of variation for determinations of MSC-specific fractions of 8.9% (*p* < 0.003), 13% (*p* < 0.006), and 25% (*p* < 0.02). The estimates of inter-site precision, 11% (*p* < 0.0001) and 26% (*p* < 0.0001), also indicated a high level of precision. Results are also presented to show the ability of KSC counting to define cell subtype-specific kinetics factors responsible for changes in the stem cell fraction during cell culture. The presented findings support the continued development of KSC counting as a new tool for advancing stem cell science and medicine.

## 1. Introduction

Kinetic stem cell (KSC) counting was recently described as a new technology to address a long-standing problem in stem cell science and stem cell medicine. To be clear, KSC counting is not a technology for counting a new type of stem cell (e.g., a “kinetic stem cell”). Instead, it is a method for quantifying any type of tissue stem cell based on cell kinetics analysis. Herein, we report a first evaluation of the precision of KSC counting, which is a critical property for assessing its potential to become an effective new tool for applications in tissue stem cell research and medicine.

KSC counting is the first described method for quantifying the specific fraction of tissue stem cells in heterogeneous tissue cell preparations isolated from diverse vertebrate organs and tissues including human [1,2]. Until the development of KSC counting, only one method was available for quantifying important human tissue stem cells. However, that method, the SCID mouse repopulating cell (SRC) assay, proved too impractical for routine usage; and it can only be used to estimate the quantity of hematopoietic stem cells (HSCs) [3,4].

In contrast, KSC counting has the potential to determine the stem cell-specific fraction of many different vertebrate tissue cell preparations. Thus far, the technology has been validated for quantifying tissue stem cells in a number of different human tissue cell preparations including HSCs in bone marrow and umbilical cord blood; liver stem cells; amniotic membrane stem cells; and mesenchymal stem cells in bone marrow and adipose tissue cell preparations [1,2]. Importantly, unlike the SRC assay, which takes at least 16 weeks to perform, KSC counting can be completed in 72 h [2].

Early in the development of KSC counting, confirming the accurate counting of tissue stem cells was recognized as a significant challenge because of the absence of valid standards of comparison besides the SRC assay [1]. Because of KSC counting’s basis on the continuation of the in vivo asymmetric cell kinetics of tissue stem cells in cell culture, initial essential validations were its ability to precisely simulate the serial cell culture kinetics of primary tissue cell preparations and its agreement with the independent quantification of asymmetrically self-renewing cells by time-lapse microscopy and cytological assays [1]. More recently, cell-specific fractionation approaches and direct comparison to the SRC assay have been used to further validate both the specificity and the accuracy of the method (cited in ref. [2]).

The present report describes the continuing development of KSC counting through a study designed to provide estimates of the precision of the method at three independent testing sites. A robust and reliable method for routine determination of the number of stem cells in tissue cell preparations would have many important impacts in stem cell science and medicine [5]. Such a method would immediately better inform basic and clinical research investigations of important human and animal tissue stem cells by allowing investigators to relate the processes, functions, and factors under study to the number of stem cells present. A more accurate measure of the dosage of stem cells in approved HSC transplant treatments would address the current pervasive problem of the CD34 count being unreliable for predicting the outcome of umbilical cord blood transplants [5,6,7] as well as underestimating the dosage of HSCs in adult donor treatments, which results in a significant number of cases of poor graft failure [8,9,10]. These stem cell-specific dosage challenges in approved HSC transplant therapies are also certainly adversely affecting the effectiveness of other stem cell types in many current clinical trials [11,12,13].

This first reported evaluation of the precision of the KSC counting method indicates a low degree of intra-site variation. Moreover, the related estimate of inter-site precision also indicates that the method can be used to reliably compare determinations of the tissue stem cell fraction and dosage performed at different sites, by different technicians, using different instruments for the required general live cell and dead cell counting [1,2]. This capability is important for the envisioned continued development of KSC counting as an important new tool for general use to improve stem cell science and medicine.

## 2. Materials and Methods

### 2.1. Oral Alveolar Bone Tissue Cell Derivation

MSC-containing cell populations derived from oral alveolar bone (aBMSCs) were isolated in accordance with the University of Michigan, School of Dentistry Institutional Review Board under approved protocol aBMSCs, IRB# #HUM00034368.

**Figure 1:**
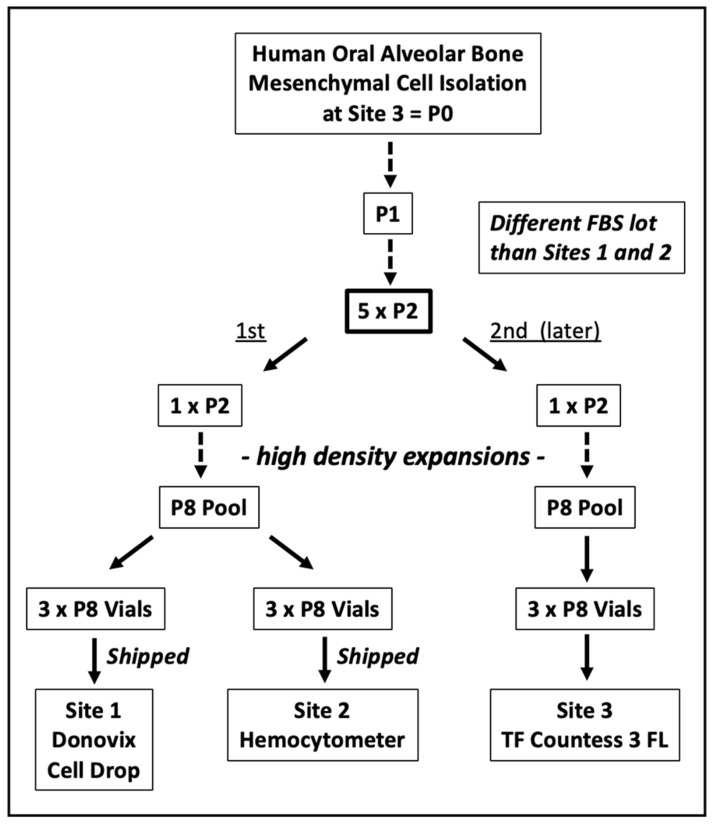
Outline of the precision study for KSC counting analyses with human aBMSC preparations. P, passage number. FBS, fetal bovine serum. One technician performed the studies at each site using the indicated cell counting instrument.

For tissue cell derivation, alveolar bone specimens were obtained from patients undergoing routine oral surgical procedures, as previously described [14]. Briefly, a 2 mm core of the alveolar bone was surgically excised, following which, approximately 0.5 cc of marrow aspirate was obtained (range, 0.1–1.5 cc). These alveolar bone marrow tissue samples were re-suspended in minimum essential medium alpha (MEMα; Gibco, Carlsbad, CA, USA) and centrifuged at 600× *g* for 10 min at room temperature. The supernatant was removed, and the cell pellet was re-suspended in complete culture medium [CCM: MEMα supplemented with 15% fetal bovine serum (FBS) along with 1% antibiotic antimycotic (Gibco), 1% L-glutamine (Gibco), and 1% L-ascorbic acid 2-phosphate (Gibco)] followed by transferring to T-25 (25 cm^2^ cell attachment area) tissue culture flasks. The flasks were left undisturbed without medium change for 5 days in a 37 °C humidified tissue culture incubator with a 5% CO_2_ atmosphere. Non-adherent cells were removed after 5 days in culture, and the medium was changed every 3 days thereafter. Once adherent cells reached 70–80% confluence, the cells were harvested by trypsinization and cryopreserved. Cell samples were later thawed and expanded as described in Section 3 (See later Figure 1) to begin serial cultures for KSC counting analyses.

### 2.2. Serial Cell Culture

Serial cell culture provides the input data required for KSC counting software analyses (see Section 2.3 below). Adherent serial cell cultures of aBMSCs were performed in triplicate in 6-well cell culture plates with 2 mL of CCM at 37 °C in humidified incubators with a 5% CO_2_ atmosphere. Briefly, 1 × 10^5^ cells/well were used to start cultures. Serial cell culture was performed as described previously for KSC counting analyses of MSC-containing tissue cell preparations [2], with the counting of live cells and dead cells at each culture passage based on trypan blue dye-exclusion. Cell counting was performed with either hemocytometer-calibrated automated electronic cell counters—Denovix (Wilmington, DE, USA) Cell Drop (Site 1); Thermo Fisher (Waltham, MA USA) Countess 3 (Site 3)—or manually with a hemocytometer slide (Site 2). Serial cell culture was conducted in CCM on the same passage schedule, which was a transfer of 1/20 of the total recovered cells every 72 ± 3 h. As required for KSC counting analyses, serial cell cultures were continued until no cells could be detected [2].

### 2.3. Kinetic Stem Cell Counting Analyses

KSC counting is not a technology for counting a new type of stem cell (e.g., a “kinetic stem cell”). Instead, it is a method for quantifying any type of tissue stem cell based on cell kinetics analysis, which has been previously described in detail [1].

The live cell and dead cell count data from parallel triplicate serial cultures were used to calculate triplicate sets of respective cumulative population doubling (CPD) data and dead cell fraction (DCF) data at each passage over the entire period of serial cell culture. The data have been deposited for free access at the Science Data Bank site 10.57760/sciencedb.10335, accession date 27 December 2023.

As described, these data were input into the TORTOISE Test^®^ KSC counting software (version 2.0) (Asymmetrex^®^, Boston, MA, USA) [2] to derive the initial stem cell-specific fraction (SCF) of each aBMSC sample evaluated. Thereafter, the SCF half-life (SCFHL) of each cell sample during subsequent serial cell culture was determined using the previously described RABBIT Count^®^ software (version 1.0) [2]. The reported mean initial cell kinetics factors including the SCF and SCFHL determinations were based on 10 independent computer simulations, as described in [2].

### 2.4. Statistical Methods

The statistical confidence of the TORTOISE Test^®^ software determinations, coefficients of variation, and the means of Pearson correlation R^2^ coefficients were evaluated by the Student’s two-tailed *t*-test with respect to a value of 0.0. For precision analyses of CPD algorithm derivations, the numerical outputs of compared algorithms were evaluated with Pearson correlation plots. The R^2^ coefficients of all pairwise comparisons of a defined set of algorithms were averaged as a basis for precision estimation. Tukey’s test was used to evaluate the confidence of inter-site variation in the initial mean SCF determinations. Pearson correlation analyses were used to evaluate the precision of the CPD algorithms. All statistical analyses were performed with 2020 GraphPad Prism 9 for macOS software, version 9.0.0.

## 3. Results

### 3.1. Intra-Site and Inter-Site Precision of MSC Fraction Determination

Previously, we have described the ability of the KSC counting computational simulation software (TORTOISE Test^®^) to use an input of cumulative population doubling (CPD) data and dead cell fraction (DCF) data from the serial culture of tissue cell samples to the determine their stem cell-specific fraction (SCF) [1,2]. Figure 1 outlines the study with nine replicate, or closely lineage-related, samples of human aBMSC isolations performed to estimate the precision of KSC counting for the determination of the SCF of tissue stem cell-containing preparations.

At Site 3, the cells for evaluation were expanded in parallel from two replicate cryopreserved vials of Passage 2 cells from the same starting donor cell preparation. From one of the expanded vials of cells, Site 1 and Site 2 received three expanded, cryopreserved, replicate sample vials by shipment on dry ice. They also received frozen aliquots of the same fetal bovine serum (FBS) lot for use in the study’s subsequent serial cell culture analyses.

Later, with the second initiating replicate vial of cells, Site 3 performed a parallel, independent expansion on the same schedule as the cells shipped to Site 1 and Site 2. However, this later second expansion used a different lot of FBS, and Site 3’s subsequent cell culture analyses were also performed with this different lot of FBS. All sites purchased and used the same commercial cell culture medium (See Section 2).

Only one technical personnel at each of the three sites, with the differences noted above, performed the serial cell cultures with live cell and dead cell counting analyses at each passage using the same standard operating procedure for serial culture and determination of the CPD and DCF data. These primary data are available at Science Data Bank DOI: 10.57760/sciencedb.10335, accession date 27 December 2023. All sites used trypan blue dye-exclusion analysis as the basis for live cell vs. dead cell quantification. Site 1 used a hemocytometer slide-calibrated Denovix Cell Drop electronic cell counter for analyses. Site 2 used a hemocytometer slide for manual counting analyses. Site 3 used a hemocytometer slide-calibrated Thermo Fisher Countess 3 FL electronic cell counter for analyses.

The responsible technician at each site thawed each of the three replicate cell cryovials per the standard operating procedures (Vial 1, Vial 2, Vial 3, respectively). Cells from each of the thawed vials were used to establish three replicate serial cell cultures (Section 2). At each culture passage (approximately every 3 days), the harvested cells were sampled three times for live cell and dead cell counting. For passaging, 1/20 of the total harvested cells were transferred immediately, before the counting, to the next culture in the series.

**Figure 2:**
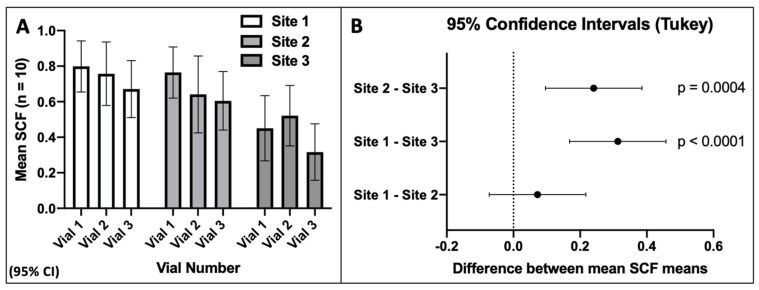
Precision analyses of KSC counting determinations of the initial mean SCF for the replicate human aBMSC samples quantified at the three different study sites. (**A**) Initial mean SCF determinations based on *n* = 10 TORTOISE Test^®^ computational simulations. (**B**) Evaluation of inter-site variation in mean initial SCF determinations based on Tukey’s test evaluating all pairwise comparisons for differences in the initial mean SCFs between two indicated sites (*n* = 6 comparisons).

The resulting KSC counting determinations of the initial mean MSC-specific fraction (i.e., the SCF) of the nine evaluated samples are presented in Figure 2A. For all nine samples, highly significant SCF determinations were obtained (*p* < 0.0001). The intra-site variation defined by the coefficient of variation for the triplicate determinations at each site were 8.9% (*p* < 0.003), 13% (*p* < 0.006), and 25% (*p* < 0.02), respectively (Figure 2A). Comparisons of the initial mean SCF determinations at each site indicated that the observed differences in the determinations at Site 1 and Site 2 values were not statistically significant. However, the observed differences in the initial mean SCF determinations between Site 3 and Site 1 or Site 2 were statistically significant (Figure 2B).

These similarities and differences were also reflected in the estimates of inter-site precision. For an analysis including only the initial mean SCF determinations of Site 1 and Site 2, the coefficient of variation was 11% (*p* < 0.0001), similar in quality to the high intra-site precision of the two sites. However, inclusion of the Site 3 initial mean SCF determinations reduced the inter-site precision to a coefficient of variation of 26% (*p* < 0.0001).

### 3.2. Evaluation of Other Initial Mean Cell Kinetics Factors

**Figure 3:**
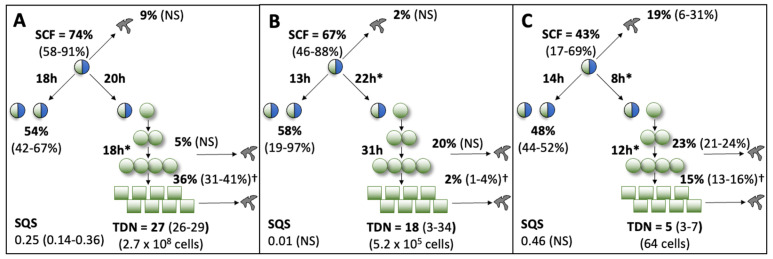
Site-specific, initial mean cell kinetics factors defined by KSC counting analyses of replicate human aBMSC samples. Each diagram depicts the underlying KSC counting cell kinetics model with the respective initial mean cell kinetics factors determined for the three replicate samples analyzed, respectively, at Site 1 (**A**), Site 2 (**B**), and Site 3 (**C**). Symmetric stem cell divisions (left) and asymmetric stem cell divisions (right) can be distinguished by their different cell products. Bivalent circles, stem cells; uniform circles, transiently-dividing committed progenitor cells; squares, terminally-arrested cells; amorphous shapes, dead cells. %, mean percent of cells of the labeled type at the start of serial culture. (#-#%), 95% confidence intervals about the mean % values. NS, not significantly different than 0.0. SCF, initial mean stem cell-specific fraction. h, indicated cell cycle times in hours. TDN, initial mean turnover over division number. (# cells), number of cells in a complete turnover cell unit. SQS, mean simulation quality score. *, indicates cell cycle times in hours that differ at a *p* < 0.05 level of confidence. †, The dead cell fraction (DCF) of terminally-arrested cells is not a KSC counting software output. It is an input based on the measured mean DCF across the entire period of serial cell culture [1,2].

In addition to quantifying the initial mean SCF of tissue cell samples, the KSC counting TORTOISE Test^®^ software estimates several additional initial mean cell kinetics factors for the stem cells, lineage-committed progenitor cells, and terminally-arrested cells that compose tissue cell preparations. Figure 3 shows diagrams of the cell kinetics model that is the underlying basis for the KSC counting computational simulation approach [1]. The diagram for each site reports the means for the indicated initial mean cell kinetics factors in the model derived from the three respective replicate samples evaluated at each site. The mean simulation quality score (SQS) at all three sites met the standard criterion of mean SQS ≤ 0.5, which gives a high degree of confidence in the initial mean cell kinetics factor determinations [1].

**Figure 4:**
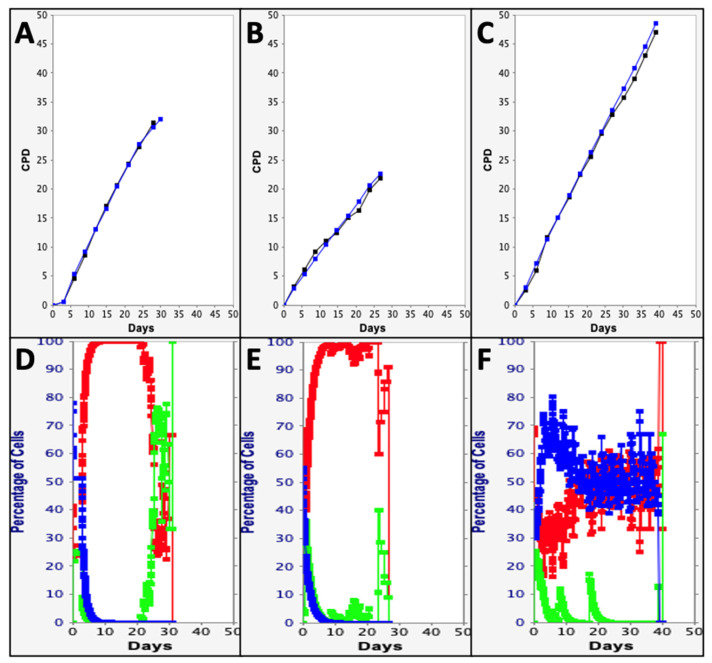
KSC counting TORTOISE Test^®^ analyses of cell subtype-specific cell kinetics profiles during serial cell culture of human aBMSC samples. Examples of computer simulations of the mean CPD data for representative replicate samples at the three different sites using the samples’ initial mean cell kinetics factors. (**A**) Site 1 (Vial 1.1; SQS = 0.22). (**B**) Site 2 (Vial 2.2; SQS = 0.01). (**C**) Site 3 (Vial 3.3; SQS = 0.16). Black lines, experimental mean CPD data for samples. Blue lines, simulated mean CPD data. y-axes, mean CPD; x-axes, days of serial culture. The fRMSE for all three presented simulations = 0.08. The fRMSE value indicates the quality of the depicted simulations’ approximation of the experimental mean CPD data. fRMSE = root-mean-squared error/maximum mean CPD value. (fRMSE is a different metric than the simulation quality score (SQS) above and in Figure 3) [1]. (**D**–**F**) Cell subtype-specific cell kinetics profiles computed with the initial mean cell kinetics factors used for the respective computer simulations (**A**–**C**). Shown are the calculated individualized cell kinetics data during passaging for stem cells (blue), transiently-dividing committed progenitor cells (red), and terminally-arrested cells (green) in terms of their percent levels.

From the data in Figure 3, it is evident that the samples evaluated at Site 1 and Site 2 were more similar to each other for their initial cell kinetics factors than to the samples evaluated at Site 3. The differences detected in the mean initial cell kinetics factors accounted for the lower initial mean SCF of the Site 3 samples and their greater rate and extent of cell proliferation (compare the experimental mean CPD data in Figure 4A–C). Samples at Site 3 had a significantly higher rate of stem cell-specific cell death (Figure 3C, 19%), which would contribute to an initial lower SCF within the three day resolution of the analysis. Site 3 samples also had shorter mean cell cycle times for both the asymmetric division of MSCs (Figure 3C, 8 h) and the transiently-amplifying division of committed progenitor cells (Figure 3C, 12 h). Both of these differences would promote higher rates and extents of cell proliferation with serial culture, as was observed (see Figure 4C, black line).

### 3.3. Evaluation of Changes in Cell Subtype-Specific Fractions during Serial Culture

The KSC counting TORTOISE Test^®^ software, (version 2.0) uses the input of the initial mean cell kinetics factors of a sample to compute the relative fractions of stem cells, committed progenitor cells, and terminally-arrested cells during the sample’s serial culture. Figure 4D–F provides examples of these computations for a representative sample at each of the three sites. Consistent with their similar initial mean cell kinetics factors, the samples from Site 1 and Site 2 had very similar profiles for changes in the fractions of the three cell subtypes during serial culture. Importantly, both showed the more typical rapid decline in the MSC-specific fraction with serial culture [1,2] (Figure 4D,E, blue trace). The rapid decline in stem cells is attributable to the rapid significant accumulation of committed progenitor cells (Figure 4D,E, red trace), which progress to producing terminally-arrested cells (Figure 4D,E, green trace) that increase in fraction later. The significantly greater turnover division numbers (TDNs) of these samples (Figure 3A,B, TDN) predict the production of large populations of committed progenitor cells and arrested cells (Figure 3A,B; 2.7 × 10^8^ cells and 5.2 × 10^5^ cells, respectively) from each asymmetric division by stem cells. The TDN is the number of generations of cell division before committed progenitor cell divisions produce terminally-arrested cells [1].

The cell subtype-specific fraction profile computations for the sample at Site 3 were quite different. The MSC-specific fraction was maintained at effectively the same level throughout the serial culture (Figure 4F, blue trace). The significantly smaller TDN of this sample (Figure 3C, TDN = 5) minimized the dilution of stem cells during the serial culture. Consistent with the smaller TDN, terminally-arrested cells increased earlier during the serial culture (Figure 4F, green trace).

### 3.4. Evaluation of the Precision of CPD Algorithms for Rapidly Counting Mesenchymal Stem Cells

Previously, we described two related mathematical algorithms that allow for rapid determination of the SCF of samples at any time during their serial culture [1,2]. Called PDT (population doubling time) algorithms and CPD algorithms, respectively, the former requires an input of 72-h PDT data and the latter requires only the input of the number of CPDs to compute the SCF of a culture. In particular, the regression analysis of CPD algorithm data yields a newly described state parameter for tissue stem cell-containing cell cultures called the stem cell-specific fraction half-life (SCFHL) [2]. The SCFHL is the number of CPDs required for the SCF to decrease by 50 percent.

**Figure 5:**
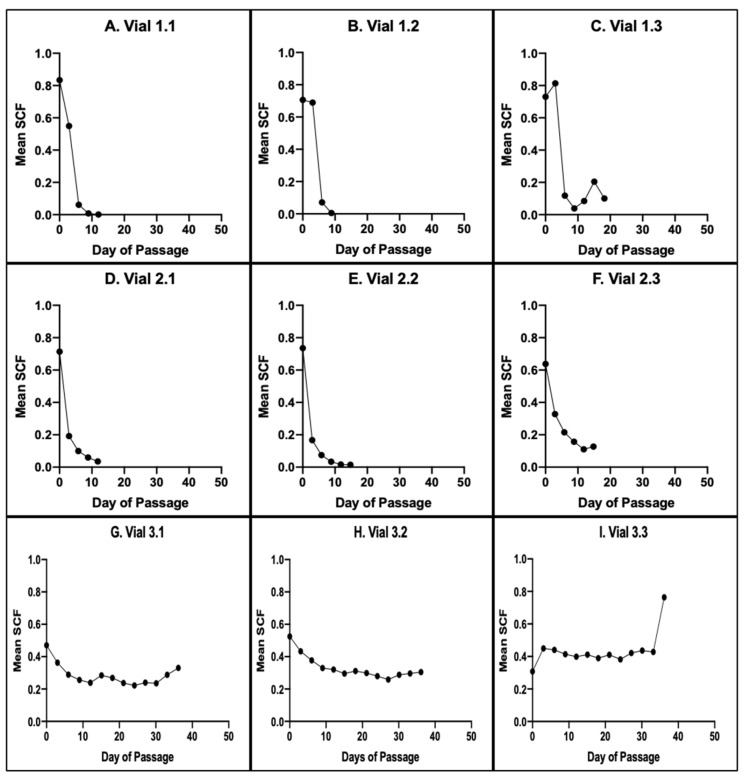
KSC counting determination of changes in the mean SCF of triplicate samples of human aBMSCs at the three independent study sites. For each evaluated sample, the mean SCF from *n* = 10 TORTOISE Test^®^ software determinations is plotted against the number of days of serial culture passages. (**A**–**C**) Site 1 replicate sample determinations. (**D**–**F**) Site 2 replicate sample determinations. (**G**–**I**) Site 3 replicate sample determinations.

CPD algorithms were derived for eight of the nine aBMSC samples in the study. These derivations were based on the mean SCF data shown in Figure 5. For each sample, the SCF determinations with serial culture from 10 independent TORTOISE Test^®^ computational simulations were averaged.

**Figure 6:**
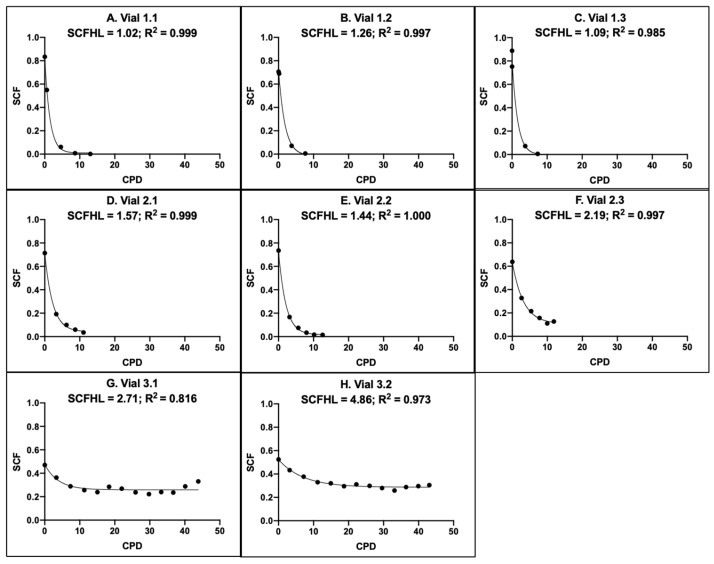
Rapid counting CPD algorithms for replicate human aBMSC samples. The mean SCF data in Figure 5 were replotted with respect to the corresponding experimental mean CPD data to derive CPD algorithms as described [2]. (**A**–**C**) Site 1 replicate sample derivations. (**D**–**F**) Site 2 replicate sample derivations. (**G**,**H**) Site 3 replicate sample derivations. Note: No algorithm could be derived for sample Vial 3.3 because no net decline in its mean SCF was detected (See Figure 5I data.).

Figure 6 shows the mean SCF output data with respect to increasing CPDs for the eight CPD algorithms that were derived. It was not possible to derive a CPD algorithm for Vial 3.3 at Site 3 because its mean SCF exhibited no net decline with serial culture (see Figure 5I).

As a first estimate of the precision of derivations of rapid-counting CPD algorithms, we evaluated the coefficients of variation for the mean of the replicate SCFHL values. As for other precision estimates, the intra-site precision for Site 1 and Site 2 was better than for Site 3. Their respective mean SCFHL coefficients of variation were 10% (*p* = 0.004) and 23% (*p* < 0.018) compared to 40% (*p* = 0.176) for Site 3. Accordingly, an inter-site analysis of the SCFHL determinations of only Site 1 and Site 2 had a coefficient of variation of 30% (*p* = 0.0004), but increased to 64% (*p* = 0.003) when the two SCFHL values from Site 3 were included.

To provide a statistically more powerful evaluation of the precision of CPD derivations, we conducted pairwise Pearson correlation analyses of the SCF output values of each CPD algorithm for 0.5 CPD increments from 0.0 to 5.0 CPD (*n* = 10). This range of CPD values corresponds to the SCF decline period of the SCFHL determinations for the Site 3 samples (see Figure 6G,H). The Pearson correlation coefficient R^2^ values obtained ranged from 0.973 to 1.000.

**Figure 7:**
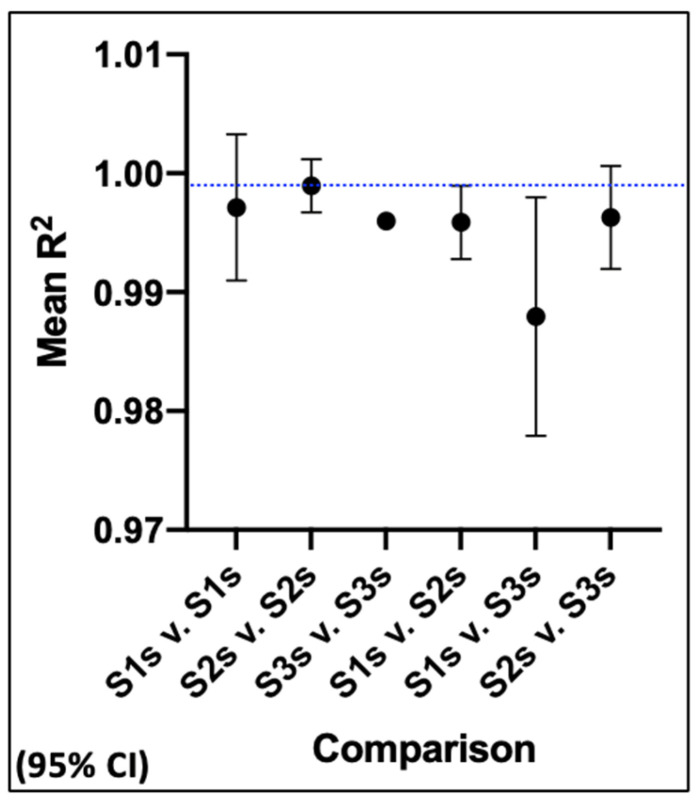
Analysis of intra-site and inter-site variation in the derivation of rapid-counting CPD algorithms. Pairwise Pearson correlation analyses were performed with the SCF outputs of CPD algorithms derived for the replicate human aBMSC samples at each site (intra) and between sites (inter). The mean Pearson correlation coefficient (R^2^) is plotted for each comparison type. Blue dotted line, highest mean R^2^ value determined. S1s, Site 1 study; S2s, Site 2 study; S3s, Site 3 study.

The mean Pearson R^2^ values for the intra-site and inter-site pairwise comparisons were evaluated. These analyses are summarized in Figure 7. The mean R^2^ values of both types of comparisons indicate a high degree of precision for CPD algorithm determinations. The mean R^2^ value for the intra-site CPD algorithm derivations at all three sites was 0.996 or greater (*p* < 0.0001). However, while three intra-site comparisons were available for Site 1 and Site 2, only one was possible for Site 3 (see Figure 6G,H). With the exception of the inter-site comparisons between Site 1 and Site 3 (mean R^2^ = 0.988; *p* < 0.0001), the inter-site precision was similarly high. The mean R^2^ value was 0.996 (*p* < 0.0001) for comparisons of Site 1 vs. Site 2 and for Site 2 vs. Site 3. These inter-site precision estimates aligned well with the differences in the SCFHLs of the samples (Figure 6).

## 4. Discussion

In a previous report, KSC counting analyses for replicate HSC-containing preparations were shown to yield statistically equivalent SCF determinations and rapid-counting algorithms (cited in ref. [2]). These previous analyses were performed in one laboratory and without a formal estimate of their intra-site precision.

The study reported herein provided a first formal estimate of the intra-site precision of the KSC counting method in three geographically distinct laboratories, also providing the opportunity for a first estimate of the inter-site precision of KSC counting. In addition, the study extended precision estimates to human MSC-containing preparations, which are a current focus for the development of many regenerative medicine therapies [11,12,13,14,15,16].

Good precision is an essential attribute for effective research and clinical tests. Because of the commonly observed high degree of biological variability, investigator variability, and technical variability associated with measurements for cellular systems, the test precision may be too low to allow for the development of reliable cellular tests. Currently, the lack of good test precision is recognized as a general problem that limits progress in regenerative medicine and cellular therapy [17]. Though infrequently estimated, the degree of inter-site precision—the ability of a test method to yield statistically equivalent results when replicates of the same cell sample are tested by different persons in different laboratories—is a particularly important consideration for establishing tests for cell product quality standards.

The presented formal estimates of the intra-site precision for KSC counting of MSCs in replicate human aBMSC samples are in keeping with the high degree of precision noted by the previous informal analyses of human HSC-containing preparations (cited in ref. [2]). Site 1 and Site 2 each tested three samples of six ideal replicates cultured in medium supplemented with the same FBS lot. However, they used different instruments for cell counting. Their respective, statistically confident, precision estimates were: 8.9% and 13% for the coefficient of variation of the mean SCF; 10% and 23% for the coefficient of variation of their mean SCFHL; and 0.997 and 0.999 for the mean Pearson R^2^ value for their CPD algorithm comparisons.

Site 3 tested lineage-related, but not ideal, replicate cell samples that were expanded and tested in medium supplemented with a different FBS lot. The intra-site precision of the Site 3 KSC counting determinations was lower. The precision estimates were: 25% for the coefficient of variation of the mean SCF; 40% for the coefficient of variation of the mean SCFHL; and 0.996 for the Pearson R^2^ value for the single available CPD algorithm comparison. Whether the known differences for the Site 3 analyses accounted for the differences in the level of precision could not be ascertained within the present study.

Because of the known differences in cell lineage and serum lot for the Site 3 KSC counting analyses, inter-site precision analyses were performed using (1) only determinations for the experimentally more similar Site 1 and Site 2; (2) determinations for all three sites; or (3) determinations for Site 3 and either of the other two study sites. The inter-site precision based on analyses for the mean SCF and mean SCFHL including only Site 1 and Site 2 was consistently better than when all three sites were included. The respective values for the coefficient of variation for the mean SCF were 11% and 26%, and they were 30% and 64% for the coefficient of variation for the mean SCFHL. In a better powered analysis of the inter-site precision of CPD algorithm derivations, which used Pearson correlation analyses, the mean R^2^ values for Site 1 vs. Site 2, Site 1 vs. Site 3, and Site 2 vs. Site 3 were 0.996, 0.988, and 0.996, respectively.

The inter-site precision analyses highlighted another powerful attribute of KSC counting, which is its ability to define differences in the overall cell proliferation kinetics of cell cultures in terms of underlying cell subtype-specific cell kinetics factors [1,2]. Consistent with their overall estimates of higher inter-site precision, the Site 1 and Site 2 samples were found to have equivalent initial mean cell kinetics factors (compare Figure 3A and Figure 3B), which yielded highly similar cell subtype fraction profiles during serial culture (compare Figure 4D and Figure 4E). The poorer precision noted in the inter-site precision estimates with Site 3 were associated with significant differences in several initial mean cell kinetics factors that translated into a dramatic increase in the stability of the SCF of the Site 3 samples. The factors and cellular mechanisms responsible for this increased SCF stability during serial culture are of significant interest. Defining them could lead to new methods for achieving consistent production of therapeutic tissue stem cells [2].

The limited analyses presented in this report are insufficient to confirm the difference in FBS lot as the cause of the increased stability of the SCF of the Site 3 samples. However, it is a very plausible explanation. In a previous report of KSC counting analyses of human adipose-derived MSC-containing preparations that differed only in growth factor supplement, FBS also conferred unusual high SCF stability during serial culture [2]. If the FBS lot is the responsible factor in the present study, the observed effects on SCF stability may account for other previously noted FBS lot variations for supporting cell proliferation [18,19,20].

The presented findings further support the case for the utility of KSC counting as a long-needed method for improving stem cell medicine and science [1,2]. The ability to quantify tissue stem cells accurately for the first time reliably, and conveniently, has many immediate beneficial applications in stem cell medicine and science. In earlier reports, KSC counting has been validated for determining the specific fraction of human HSCs in several different preparations used for both stem cell research and stem cell medicine [1] (cited in ref. [2]) and for defining the effects of growth-promoting and toxic agents on MSCs [2] and HSCs [1]. These capabilities have significant potential to provide a solution to the continuing problem of unknown insufficient dosage in HSC transplantation therapies [7,8,9,10], enable better optimization of stem cell-dependent tissue engineering and cell biomanufacturing, and allow for the earlier detection of drugs that fail late in clinical trials or post-marketing because of chronic organ failure due to tissue stem cell toxicity.

## 5. Conclusions

A three-site inter-lab study was conducted to estimate the precision of kinetic stem cell (KSC) counting determinations of the specific fraction and cell culture kinetics of mesenchymal stem cells (MSCs) present in a heterogeneous preparation of human oral alveolar bone-derived mesenchymal tissue cells. The obtained estimates of both intra-site and inter-site precision indicated that KSC counting had precision that was sufficiently high to serve as an effective test for MSC quantification for research, medicine, and drug evaluation applications. In addition, the study detected a significant increase in the stability of the fraction of MSCs during serial culture that was associated with a difference in the lot of fetal bovine serum used for culture medium supplementation.

## Data Availability

All primary CPD data and DCF data are deposited for free access in Science Data Bank at DOI: 10.57760/sciencedb.10335, accession date 27 December 2023.
